# Ordering motivation and Likert scale ratings: When a numeric scale is not necessarily better

**DOI:** 10.3389/fpsyg.2022.942593

**Published:** 2022-09-23

**Authors:** Yulia Tyumeneva, Yulia Sudorgina, Alexandra Kislyonkova, Maria Lebedeva

**Affiliations:** ^1^Institute of Education, HSE University, Moscow, Russia; ^2^Laboratory for the Neurobiological Foundations of Cognitive Development, HSE University, Moscow, Russia; ^3^Center for Cognition and Communication, Pushkin State Russian Language Institute, Moscow, Russia

**Keywords:** transitivity, ordinal structure, motivation, rating scale, pairwise ordering

## Abstract

Measuring psychological attributes, such as motivation, typically involves rating scales, assuming that an attribute can be ordered, and that ratings represent this order. Previously, only the first assumption had been tested, albeit limited. First, we checked the ordinal structure of motivation, looking at whether people can establish transitive relations between motivation levels in pairwise comparisons; and we found different ordering patterns: strict transitive, weak transitive, changing order, and intransitivity. The rate of intransitivity was similar to that found previously and was somewhat higher than we obtained when we asked participants to compare definitely quantitative attributes (such as weight). Second, we checked if specific ordering patterns were related to individual interpretations of the statements that deviated from expected motivation types. Indeed, about a third of participants miscategorized statements, and these deviant interpretations were related to intransitivity as well as weak transitivity. Third, we checked whether Likert ratings represent the order of motives obtained from pairwise comparisons. We found rather homomorphic representation: ratings correlated with the order, but they did not differentiate between different ordering patterns and hierarchies of motives. We conclude that the Likert rating scale provides less information about respondents than pairwise ordering. The findings question the mainstream practice of using rating scales without testing underlying assumptions.

## Introduction

The question about the additive vs. ordinal structure of psychological attributes concerns the very basic assumptions of psychometrics. However, this assumption is rarely explored experimentally. The lack of such explorations can be mostly explained by fundamental difficulties with the experimental control of extraneous factors in psychological research, which is crucial for establishing an attribute structure (Trendler, 2009). Another explanation is that psychometrics typically proceeds by modeling the structure of the test, not trying to explore what the underlying psychological attribute is. That is why good theories about attributes and test-taking behavior are still absent despite reports of the greatest need for them (Kyngdon, 2013; Rhemtulla et al., 2015; Uher, 2021).

Here, we addressed the most basic assumption underlying measurement in psychology, namely, the ordinal structure of psychological attributes. The ordinal structure is confirmed if any three levels of an attribute meet the conditions of transitivity, asymmetricity, and connectivity. For example, to be transitive, any of the levels of an attribute a, b, and c must satisfy the following condition: if a > b and b > c, then a > c; otherwise, there are transitivity violations that prevent ordering levels of the attribute, and the attribute cannot be treated in the ordinal scale. Moreover, since the ordinal structure is a prerequisite for quantitative structure, the failure to meet the order conditions makes the attribute immeasurable on the interval scale (Michell and Ernst, 1996).

A few studies on the ordinal structure of psychological attributes have shown that most people generally are able to order attributes in triads using pairwise comparisons (a > b, b > c, a > c) without transitivity violations (Michell, 1998; Morris et al., 2017; Tyumeneva and Vergeles, 2021). It is important, that despite different methods used, the studies showed a surprisingly consistent results, namely, low rate of transitivity violations (5–8% of all comparisons) and their uneven distribution.

However, due to the small number of such studies little is known about the sources for intransitivity. Some researchers have found that transitivity is violated when stimuli require more cognitive effort (e.g., the larger amount of pair comparisons and semantic complexity of the statements) (McGrane, 2009) or embedded in different contexts (Johnson, 2001). Besides, individual interpretations of statements were found to deviate from interpretations commonly expected; moreover, these individual interpretations interfered with ratings independently of the strength of the attribute measured. That is, if one understands a statement differently, his/her rating of this statement will reflect not only agreement/disagreement, but also some personal characteristics (Arnulf et al., 2020). Given that ordering attributes requires respondents to unambiguously understand and distinguish paired statements, any deviation of individual interpretation from expected one can intrude ordering and hamper transitivity. But to our knowledge previous research had not explored effects of individual interpretation on transitivity.

Therefore, this study was driven by the need to replicate findings on transitivity, and in addition, to test effects of individual interpretations of statements on ordering and transitivity.

There is also another, almost unexplored, question regarding the Likert scales commonly used to rate psychological attributes. Given that some people fail to preserve transitivity when ordering attributes, how does this intransitivity affect their Likert ratings? Or, if people judge attributes as equal in pairwise comparisons, how do Likert ratings reflect this? We found only one paper on the transitivity of work values where these questions were partially explored (Ravlin and Meglino, 1989). The authors showed that intransitivity was associated with more similar Likert ratings for those values which were not transitively ordered. However, it is not clear whether numerical similarity in ratings reflects different patterns of ordering such as intransitivity, weak transitivity (a > b ≥ c, then a ≥ c) or changing order (when a person preserves transitivity but not the same order across triads). Also, it is not quite clear, whether Likert scale could indicate cases where transitivity was violated. Given that the numerical similarity between ratings reflected intransitivity, how selective were this similarity values? Was it possible to identify any threshold values in the numerical similarity of ratings which necessary and sufficient to detect intransitivity? It would be of practical utility to have some indicators in the Likert scale which could help identify cases where the ordinal structure of attributes was not established.

In the current research, we focus on motivation, a psychological attribute that is typically assessed with Likert scales. The main idea of this study, besides the exploration of motivation ordinal structure, is to explore if and how Likert ratings reflect possible patterns of ordering motivation. Specifically, the study aimed to answer the following questions: (1) To what extent will previous results on the intransitivity be reproducible on new data? (2) To what extent can intransitivity be attributed to individual misinterpretations of statements? (3) How do the patterns of ordering motives (including intransitivity) and individual misinterpretations correspond to Likert ratings of these motives?

## Materials and methods

To our knowledge, two methods of testing transitivity have been used. One is the binary tree procedure based on the unfolding theory (Coombs, 1950). It constructs ordered sets of statements regarding the same attribute, but differing in terms of the attribute intensity level (say, from A to F). An intransitivity occurs when one judges Statement B closer to one’s own attitude than Statement C, C closer than D, but judges D closer than B (Michell, 1998). Another method uses pairwise comparisons of three statements, each representing some attribute, say A, B, C (such as different motives or persons) (Morris et al., 2017; Tyumeneva and Vergeles, 2021). An intransitivity occurs when one prefers attribute А to attribute B, B to C, but C to A.

The binary tree procedure is more demanding since respondents deal not just with the semantics of the attribute (say, an attitude to homosexual marriage) but also with the semantics of the descriptions of the attribute levels (extremely support vs. support but not encourage). The procedure of pairwise comparisons is a less laboring method, as respondents deal only with the semantics of the attribute. Moreover, since we were interested in how the Likert scale can identify intransitivity and other ordering patterns, it was more practical to take ready-made statements from a well-established Likert scale, than to construct new statements for the binary tree procedure. So, here the method of pairwise comparison of attributes was used.

### Participants

Two hundred and fifty-two participants (211-females, *M* = 20.9 years, *SD* = 3.5) participated in the study. They were university students who got extra points as a reward for participation.

### Materials and procedures

The study was conducted online *via* the Online Test Pad platform.^1^

We used statements from the Academic Motivation Scales questionnaire (AMS, Vallerand et al., 1992) in its adapted version (Gordeeva et al., 2014) about internal and external motivation as well as amotivation.

Internal motivation (I):

*I am interested in learning* (I1).

*I just like to learn and learn new things* (I2).

*I really enjoy learning new materials during classes* (I3).

External motivation (E):

*I have no choice*, *otherwise I will not be able to have a comfortable livelihood in the future* (E1).

*I have no choice*, *because class attendance is being marked* (E2).

*To avoid problems with the study office and exams* (E3).

Amotivation (A):

*I have got used to going there*, *but honestly*, *I do not exactly know why I do it* (A1).

*I once had good reasons for going to university; however*, *now I wonder whether I should continue* (A2).

*Honestly*, *I do not know; I really feel that I am wasting my time at university* (A3).

Each statement from one motive was paired with each statement from two other motives, so, there were 27 pairs of statements. The order of statements in each pair was random.

First, participants compared the statements in pairs and chose one which better explains why they go to study. The participants could tick off “equally important” if they could not choose between motives.

For example, a pair of motives were presented as follows:

*I attended classes last week because…*

- *I am interested in learning*.
- *To avoid problems with the study office and exams*.

*Equally (if you really cannot choose one of the reasons)*.

The participants could not change their answers or return to the previous pairs after they gave an answer.

Second, participants were asked to evaluate the same nine statements using a 5-point Likert scale (from 1-completely disagree to 5-completely agree).

Third, in order to check whether specific ordering patterns were related to individual interpretations of the statements that deviated from expected motivation types, we asked the participants to relate each statement to one of three types of motivation. Motivation types were described for the participants as follows:

*Internal motivation-a person aims to learn new things and enjoys the process of studying*.

*External motivation-a person aims to avoid problems right now or in the future and get some benefits from the study*.

*The absence of motivation*, *or amotivation-a person goes to study because he/she has got used to it*, *but does not see any sense in it*.

In addition, we attempted to establish the “lowest” rate of intransitivity which we could expect even for definitely quantitative attributes. Therefore, in addition to the main procedures, we have asked participants to order well-defined attributes, such as age, height, and weight (Morris et al., 2017). If some particular rate of violations can be found when ordering definitely quantitative attributes, it would be reasonable to expect that for ill-defined attributes, such as motivation, this rate cannot be lower. The procedure was the same: participants made a set of pairwise rankings in which they pairwise compared people well-known for them (best friend, mother, and myself) and chose one person who possessed a certain characteristic more than another person (i.e., who is higher, who weighs more or who is older). They could also use the “equally” option. Participants compared all possible combinations of three people and three characteristics.

### Analytical strategy

To assess the relation of specific ordering patterns with individual interpretations of the statements that deviated from expected motivation types, we used a chi-square test with a 95% CI as a threshold.

To estimate the relation between the Likert ratings and subjective similarity of motives, we first calculated the differences between the totals on each Likert subscale, thus receiving a subjective distance between motives. Then we summed the number of times the “equally important” option was selected for a respective pair, internal/external, etc., for each individual. Then we calculated Pearson’s correlations between the subjective distance and the number of the “equally important” using a 95% CI.

To assess the effect of the ordering patterns on between-motives differences in Likert scores we used a between-group one-way analysis of variance with a 95% CI.

## Results

Firstly, we conducted quality control to remove participants who might not take the task seriously. We checked how participants ordered people by age. There were four people who rated themselves as older than their mothers, and one participant assessed his/her and mother’s age equally. We excluded these participants from the analysis. As a result, 247 participants were left for further analysis.

### The distribution of transitivity violations

The pairwise comparisons resulted in a triad, e.g., I1E2-E2A3-I1A3, in such a way that we could check transitivity, e.g., I ≥ E ≥ A, and I ≥ A. For each of the 252 participants, we analyzed 27 triads. Overall, there were 2,223 triads, amongst which 178 (8%) were intransitive. This 8% of intransitive triads were made by 80 participants (32.3% of the sample). Around 68 percent of participants (*n* = 167) showed only transitively ordered motives responses.

By contrast, when the participants ordered age, height and weight, that is, the attributes with definitely quantitative structures, they showed 2.6% of violations on average (with maximum 4% for weight). In other words, if we have 2–3% of transitivity violations with undeniably quantitative attributes, then 8% of violations obtained for the motivation attribute is not quite large.

### Ordering patterns and individual interpretations of statements

Participants demonstrated different ordering patterns: (1) “strict transitive” who did not use the “equally” option at all and did not change the order of motives across triads (11%); (2) “weak transitive” who resided on the “equally” option at least once (46%); (3) “changing order” who ordered motives differently across triads, still preserving strict transitivity in each triads (11%); (4) “intransitive” (32% with at least one violation).

What was the role of individual interpretations of the statements in different ordering patterns? Namely, we explored the effects of the deviation of individual interpretation of some statements from the commonly expected interpretation. When the participants were asked to relate each statement to one of three types of motivation, a total of 93 people (38%) misclassified the statements. These “deviant” interpretations were related to weak transitivity and intransitivity, but not to the “changing order” pattern [χ^2^(3, *N* = 193) = 6.52, *p* < 0.01]. In other words, participants interpreting statements differently were more likely to violate transitivity or resort to weak order, than change order.

### Likert ratings and ordering patterns

**Figure 1:**
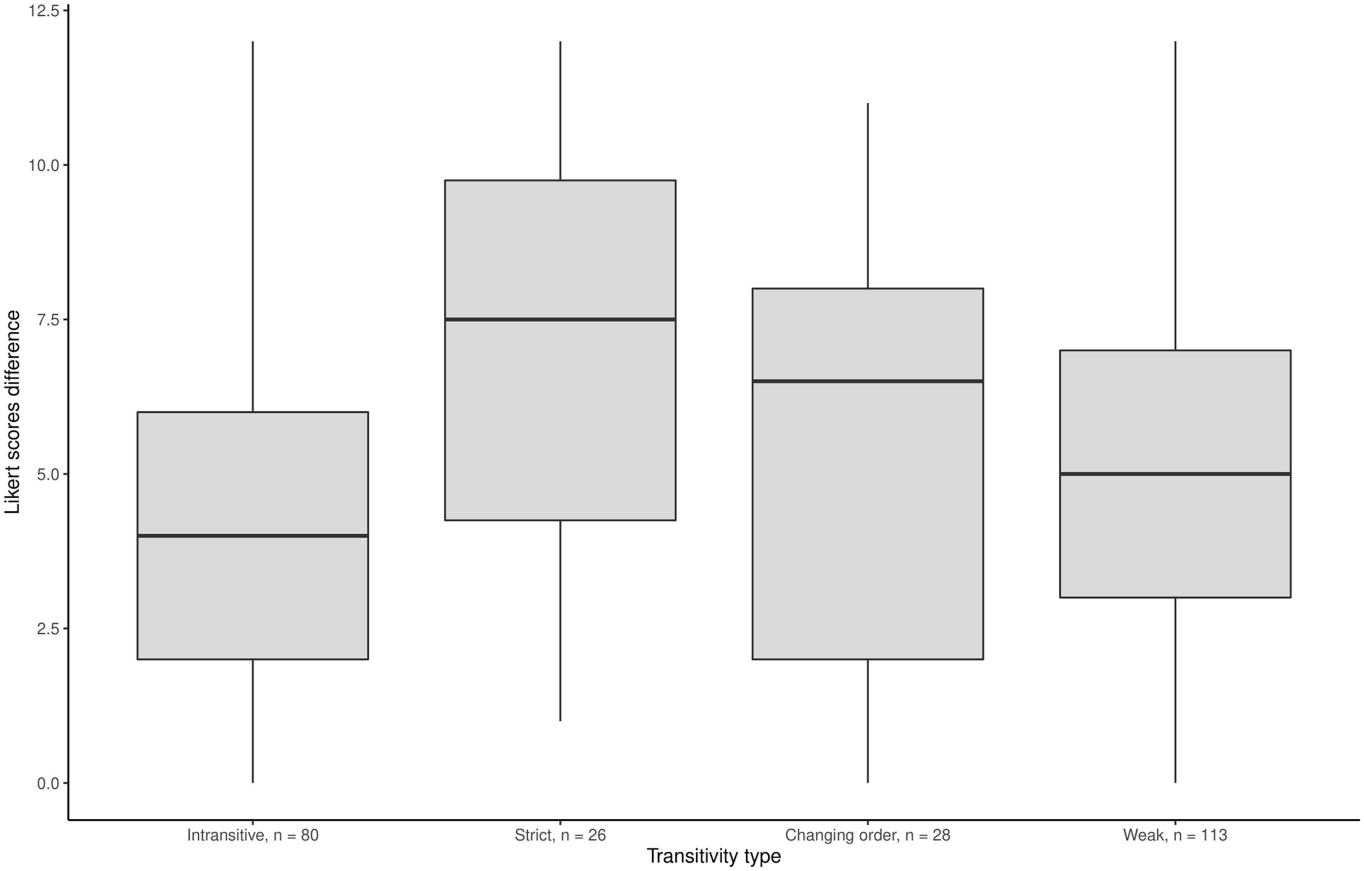
Mean difference in Likert scores between internal and external motivation scales.

**Figure 2:**
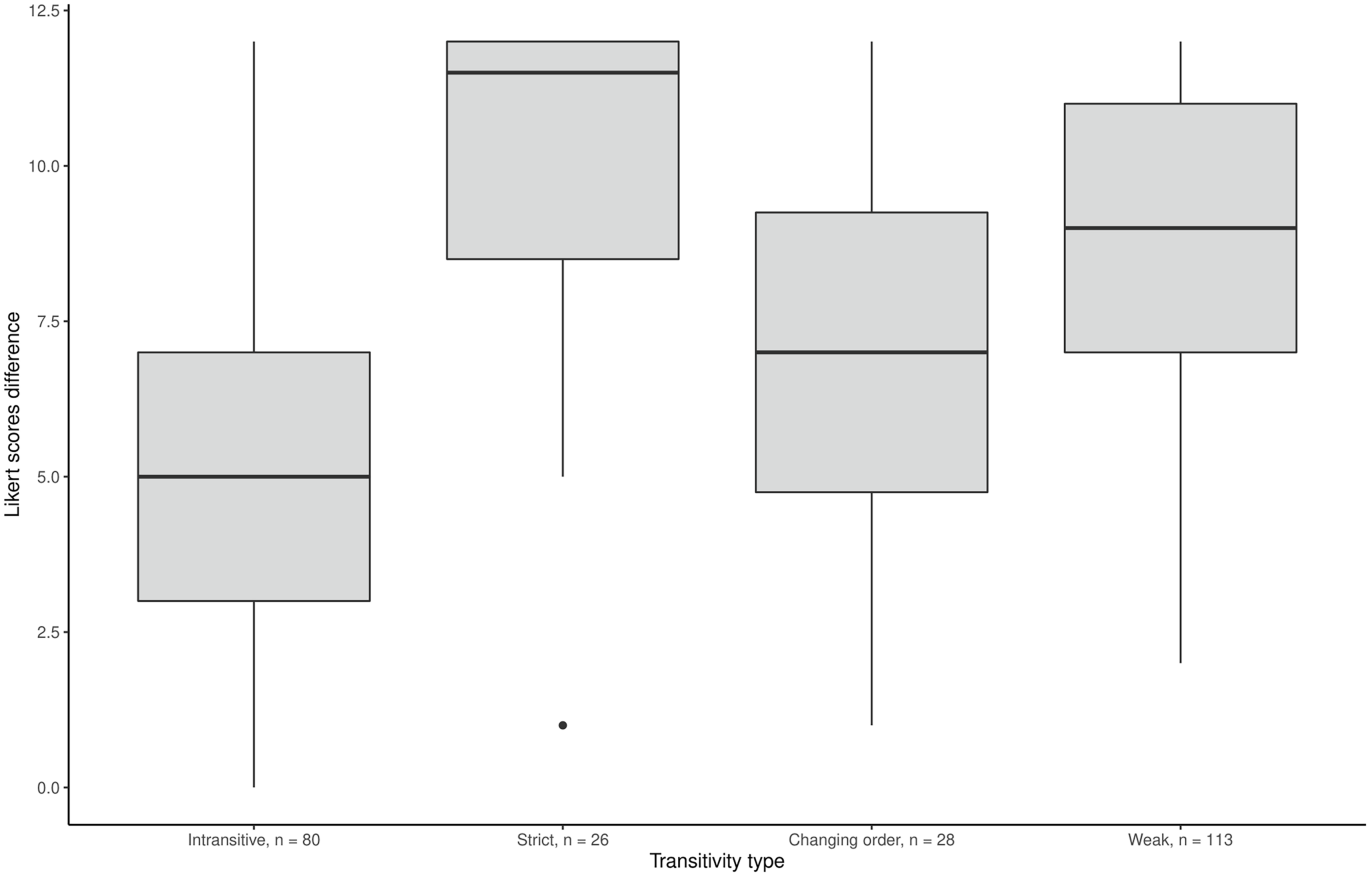
Mean difference in Likert scores between internal motivation and amotivation scales.

**Figure 3:**
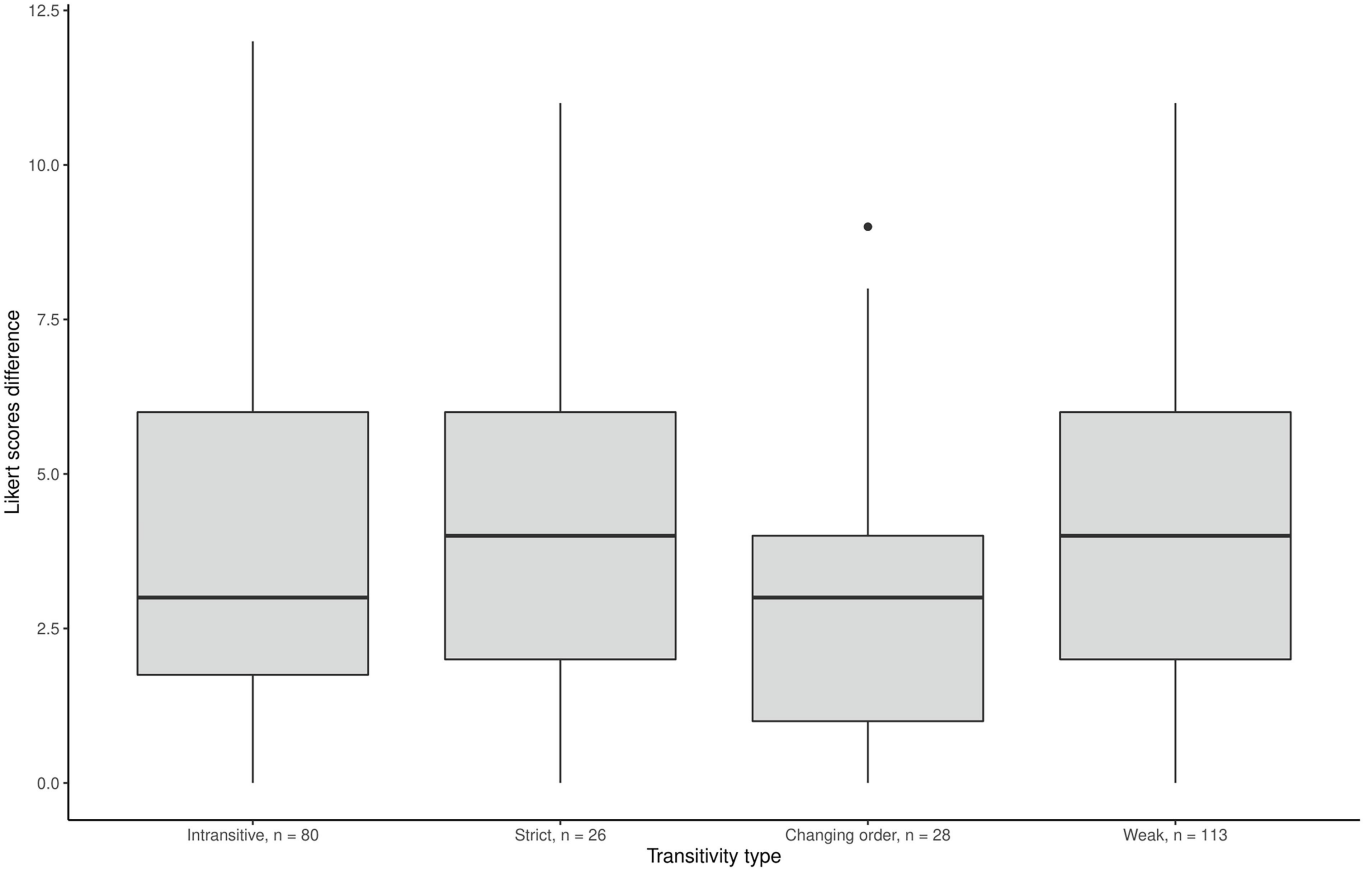
Mean difference in Likert scores between external motivation and amotivation scales.

We could expect that the biggest between-motives differences in Likert scores might come from “strict transitive” individuals rather than from “weak” or intransitive ones. Indeed, the results were partly in line with this reasoning (Figures 1–3) as the effect of ordering patterns on the difference between Internal motivation and amotivation was significant, *F*(3,243) = 29.04, *p* = 0.001. *Post hoc* analyses using the Bonferroni adjustment indicated that the average Likert score difference was significantly lower in the intransitive group than in the other three groups (see Figure 2). Additionally, we found the correlation −0.53 (*p* < 0.001) between the number of the “equally” options chosen in pairwise comparisons and the between-motive differences in ratings: the more often the two motives were considered equal, the less was the difference between these motives on the Likert scale.

**Figure 4:**
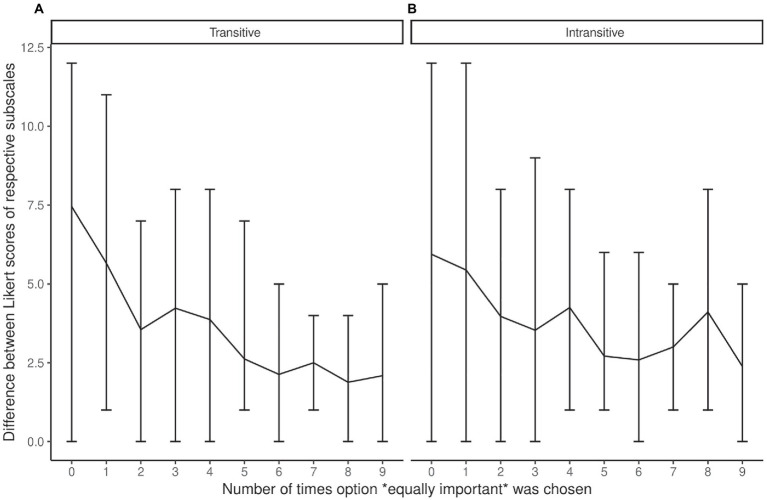
**(A,B)** Between-motives difference of Likert scores for motives estimated as “equally important” for “transitive” and “intransitive” participants (the central line connects mean values; the error bars represent the range of between-motives difference).

However, when we checked whether there was a minimal difference between the ratings of two motives which could guarantee that the participant used the “equally” option or made an intransitive triad at least once, we found no such between-motives differences (Figure 4). As you can see, the numeric difference between the motives on the Likert scale could be the same for any ordering patterns.

### The hierarchy of motives in Likert ratings and in pairwise comparisons

For transitively ordered motives, it was possible to derive individual hierarchies, that is, to say that one motive is dominant and the another is subordinate (for strict transitivity) or that at least one motive is dominant or subordinate (for weak transitivity). The question was, how did Likert ratings correspond with these hierarchies?

For each participant, we compared the hierarchy of the motives derived from triads with the hierarchy of the respective motives derived from the Likert scale. We found almost perfect correspondence: the ratings failed to reflect pairwise hierarchies only in 3.8% of cases. Thus, the Likert scale mostly represents the ordinal relations obtained in pairwise ordering. However, the inverse operation was not possible: the Likert scale provided no information about what exactly hierarchy was obtain in pairwise ordering. For example, Likert scores 15, 13, 11 (for I, E, A, accordingly) could correspond with a full hierarchy I > E > A. But the same Likert scores could just as well correspond with a partial hierarchy where only a dominant I motive or only subordinate a motive was identified. This is because Likert ratings did not distinguish between strict and weak transitivity, as well as intransitivity. In other words, the Likert scale provided less information about motivation than pairwise comparisons did.

### Likert ratings of statement interpretations

We did not find any indicators in Likert ratings that could be associated with participants who misclassified the statements across motivation types. There was no statistically significant difference in Likert scores between those who misclassified and those who did not (for all pairs of motives *p* > 0.30). Also the correlation between the number of the “equally” option chosen and between-motives differences in Likert ratings was practically the same as for participants who misclassified and for those who classified correctly (ranging from *r* = −0.50 to *r* = −0.35 (*p* < 0.001 for each correlation for different pairs of motives)).

## General discussion

In this work we pursued two main goals. First, to explore associations of ordering patterns, the subjective similarity between motives, and individual interpretations of statements; and second, to explore how these patterns were represented by the Likert scale.

Given that in the current research the distribution of intransitivity was similar to what had been found in another work (Tyumeneva and Vergeles, 2021), we incline to think that our results reflect some general regularities. With this in mind, we discuss our results.

We found that the participants preserve transitivity in various ways, such as strictly ordering motives, changing the order of motives in triads (albeit preserving strict order in each triad) or treating motives as similar. Usually, different ordering patterns, as well as intransitivity, are explained by subjective similarity of motives. For example, if the subjective difference between motives is large, then the individual can order them strictly. However, if the difference is small, then the individual can either equate the motives, change their order, or violate transitivity (Ravlin and Meglino, 1989; Michell, 1998; Tyumeneva and Vergeles, 2021).

Without refuting this explanation, we allow for another. Namely, the individual may consider statements in a pair as belonging to the same motive. This would force the individual to use the “equally” option or violate transitivity. But in this case, the subjective similarity would not concern the strength of motives, but their essence; they would be perceived as identical. At least insofar as the statements that represent the motives are perceived as identical.

Trying to prevent this problem, we paired statements belonging to different motives and relied on their semantics and on empirical data about their factor structure (Ryan and Deci, 2000; Gordeeva et al., 2014). In spite of this, about a third of the participants interpreted the statements in a different way. It seems we are faced with a well-known problem when the structure of attributes found at the inter-individual level is not reproduced at the intra-individual level (Feldman, 1995; Mischel and Shoda, 1998; Molenaar et al., 2003). The effect of individual interpretations of statements on ratings has already been shown (Arnulf et al., 2020); our results showed this effect on the necessary condition for ratings, transitivity, as well.

Nevertheless, the “changing order” pattern seems not to be affected by individual interpretations. We can only speculate that this pattern can be caused by the instability of the individual motivation hierarchy. A similar conclusion can be found in decision making research, where it has been shown that people change their minds during self-reporting and thus do not show stable preference, yet preserving transitivity (Cavagnaro and Davis-Stober, 2014). It should be noted however, that as the “changing order” pattern was underrepresented with only 11%, so, the statistical test might not be able to detect the relations with certainty.

Regarding the second of our main questions (how the Likert scale represent the hierarchy of motives obtained from pairwise comparisons) we found that the Likert ratings did correlate with the hierarchies, but they provided significantly less information regarding an individual than pairwise comparisons did. In particular, the ratings did not distinguish (1) a complete hierarchy of motives, when all motives were strictly ordered, from (2) a partial hierarchy, when the position of only some motives could be determined, and from (3) intransitivity, when a respondent could not transitively order the motives.

Technically, the scale’s poor distinguishability can come from its limited sensitivity to the level of subjective similarity between motives. Although between-motives differences in ratings corresponded with how often participants equated the particular motives in pairwise comparisons (see also Ravlin and Meglino, 1989), we did not find thresholds for between-motives differences which could separate participants who reported similarity between motives vs. those who did not. It was because of the absence of thresholds that it was impossible to identify participants with any particular patterns.

The absence of thresholds for between-motives differences could be due to the length of the scale limited to only five points. If the scale was longer (say, seven or nine), this could perhaps lessen the problem, but it would require respondents to use the numerical scale consistently. It is also possible that a limited range of total scores for each motive, in our version including only three statements, caused the poor distinguishability of the scale. If so, increasing the number of statements could lead to a more nuanced total scores, which perhaps could help establish thresholds for between-motives differences at least for intransitivity cases.

Furthermore, it is worth noting that we chose quite strict criteria to define patterns, e.g., one intransitive triad was enough to consider a participant intransitive. We did so based on the unambiguity of such criteria, and because there were no other established rules. But we recognize the need for more elaborated grounds for these kinds of decisions and further research in the area of ordinal judgments.

All in all, this study should be viewed as a part of a critical discussion on the transition to quantitative scales as supposedly more informative (Michell, 1999, 2003, 2012; Molenaar, 2004; Grice et al., 2012). In fact, as our findings showed, the ordinal scale (even very basic pairwise ordering) can give more information than numerical measures, especially when applied to the attribute the quantitative structure of which questionable.

## Data availability statement

The raw data supporting the conclusions of this article will be made available by the authors, without undue reservation.

## Ethics statement

Ethical review and approval was not required for the study on human participants in accordance with the local legislation and institutional requirements. The patients/participants provided their written informed consent to participate in this study.

## Author contributions

YT conceived the presented idea, developed the research design, and wrote the manuscript. YS and AK developed the research design, obtained and analyzed the data, and wrote the manuscript. ML conducted data collection and wrote a part of the manuscript. All authors contributed to the article and approved the submitted version.

## Funding

This work is an output of a research project implemented as part of the Basic Research Program at the National Research University Higher School of Economics (HSE University).

## Conflict of interest

The authors declare that the research was conducted in the absence of any commercial or financial relationships that could be construed as a potential conflict of interest.

## Publisher’s note

All claims expressed in this article are solely those of the authors and do not necessarily represent those of their affiliated organizations, or those of the publisher, the editors and the reviewers. Any product that may be evaluated in this article, or claim that may be made by its manufacturer, is not guaranteed or endorsed by the publisher.

## Footnotes

^1^ https://onlinetestpad.com
